# Posterior urethral stenosis: a comparative review of the guidelines

**DOI:** 10.1007/s00345-022-04131-y

**Published:** 2022-08-26

**Authors:** Behzad Abbasi, Nathan M. Shaw, Jason L. Lui, Kevin D. Li, Architha Sudhakar, Patrick Low, Nizar Hakam, Behnam Nabavizadeh, Benjamin N. Breyer

**Affiliations:** 1 Department of Urology, University of California San Francisco, San Francisco, USA; 2 Department of Urology, MedStar Georgetown University Hospital, Washington DC, USA; 3 Department of Epidemiology and Biostatistics, University of California San Francisco, San Francisco, USA

**Keywords:** Urethral stricture, Urethral stenosis, Posterior, Bladder neck contracture, Vesico-urethral anastomotic stenosis, Pelvic fracture urethral injury

## Abstract

**Purpose:**

We aimed to provide a thorough comparative review of the available guidelines on the diagnosis, management, and follow-up for patients with posterior urethral stenosis by the American Urologic Association (2016), Société Internationale d’Urologie (2010), and European Urologic Association (2022).

**Methods:**

The AUA, SIU, and EAU guidelines were evaluated for recommendations on the diagnosis, evaluation, and treatment of posterior urethral stenosis. We also included the EAU and AUA urologic trauma guidelines for the trauma-related stenosis. The level or strength of recommendations is included in case of disparity between the guidelines.

**Results:**

The three guidelines align considerably in recommendations provided for the diagnosis, management, and follow-up of patients with posterior urethral stenosis. SIU and EAU emphasize the role of repeat endoscopic treatment in guidelines compared to AUA.

**Conclusion:**

The preferred method to repair bulbo-membranous stricture/stenosis following radiation therapy remains an area of active interest, focusing on continence preservation. Additionally, there may be a role for advanced endoscopic treatments with or without adjunct therapies to manage even obliterated stenoses.

**Supplementary Information:**

The online version contains supplementary material available at 10.1007/s00345-022-04131-y.

## Introduction

Urethral stricture is an abnormal narrowing of the urethra resultant from fibrosis in the spongiosus tissue surrounding the anterior urethra [1]. In the posterior urethra -extending from bladder neck to membranous urethra- a ‘stenosis’ is the preferred term because the segment lacks spongiosal tissue [1, 2]. The majority of urethral stricture occurs in the anterior urethra (92%), leaving the posterior stenoses relatively understudied [3].

Posterior urethral stenosis may occur iatrogenically, most notably due to treatment for benign prostatic hypertrophy (BPH) and prostate cancer [1, 2]. Pelvic fracture urethral injuries (PFUI) represent a well-described traumatic etiology for posterior urethral stenosis. Based on etiology and current patient anatomy, posterior urethral stenosis is treated with various procedures ranging from catheterization to abdominoperineal reconstruction.

Formerly, Société Internationale d’Urologie (SIU) and American Urologic Association (AUA) had published guidelines on the evaluation, management, and follow-up for urethral strictures, respectively in 2010 and 2016 [4, 5]. Similarly, the European Urologic Association (EAU) recently published guidelines for evaluating and managing posterior urethral stenosis [6]. Our group had previously published a review article to compare the guidelines provided by AUA and SIU on urethral stricture and stenosis [7]. With updated guidelines, we aimed to provide an in-depth review to compare the AUA, SIU, and EAU guidelines for assessment, management, and follow-up of patients with posterior urethral stenosis. This work is the second of this series, preceded by a review article comparing the latest guidelines on anterior urethral stricture [8].

## Methods

The SIU, AUA, and EAU guidelines were evaluated for recommendations on the diagnosis, evaluation, and treatment of posterior urethral stenosis. For the trauma-related stenosis, we additionally included the EAU and AUA urologic trauma guidelines as, unlike the SIU urethral stricture guideline, the EAU and AUA stricture guidelines did not provide suggestions for the diagnosis and management of trauma-related urethral injuries [9, 10].

The level or strength of recommendations is included in case of disparity between the guidelines. The EAU and SIU guidelines have adopted the Oxford classification system, while AUA has developed a distinct evidence-grading system [7]. SIU stratified their recommendation strength from A to D based on the level of evidence [4]. The EAU guidelines recommendations are based on a modified GRADE methodology considering the level of evidence, the magnitude of effect, certainty of the results, the balance between desirable and undesirable outcomes, and the impact/certainty of patient values and preferences [6, 11, 12]. AUA approaches the strength of the recommendations based on evidence strength, certainty level, the magnitude of benefit or risk/burdens, and the Panel’s judgment regarding the benefits and risks/burdens [5] (see appendix).

## Non-traumatic posterior urethral stenosis

Posterior urethral stenosis can result from iatrogenic causes, including trans-urethral resection of the prostate (TURP) for BPH, radiation/high energy therapies, or open/robotic surgeries to treat prostate cancer [1, 2]. Treatments for non-traumatic posterior stenoses include conservative management as well as endo-luminal and (minimally) invasive interventions [6]. Optimal treatment and timing, however, are individualized based on the patient’s situation.

The pre-operative evaluation should include a history, physical examination, and post-void residue (PVR) measurement [SIU: A]. Urine analysis/culture and sensitivity/leukocyte esterase screening test, as well as blood urea nitrogen, creatinine, glucose, and PSA measurement, could be used adjunctively [SIU: B]. Cystoscopy is currently considered the optimum imaging modality for pre-operative evaluation of posterior stenoses, which could guide treatment [SIU: B]; particularly in case of a combination of anterior stricture and posterior stenosis. Further, urodynamics could be used to evaluate voiding dysfunction and/or urinary incontinence [SIU: C].

Recommendations for iatrogenic, non-traumatic posterior urethral reconstruction treatment are based on the etiology and the location of stricture/stenosis.

### Posterior urethral stenosis following iatrogenic injury (e.g., post-TURP)

#### Bladder neck contracture (BNC)

**Fig. 1:**
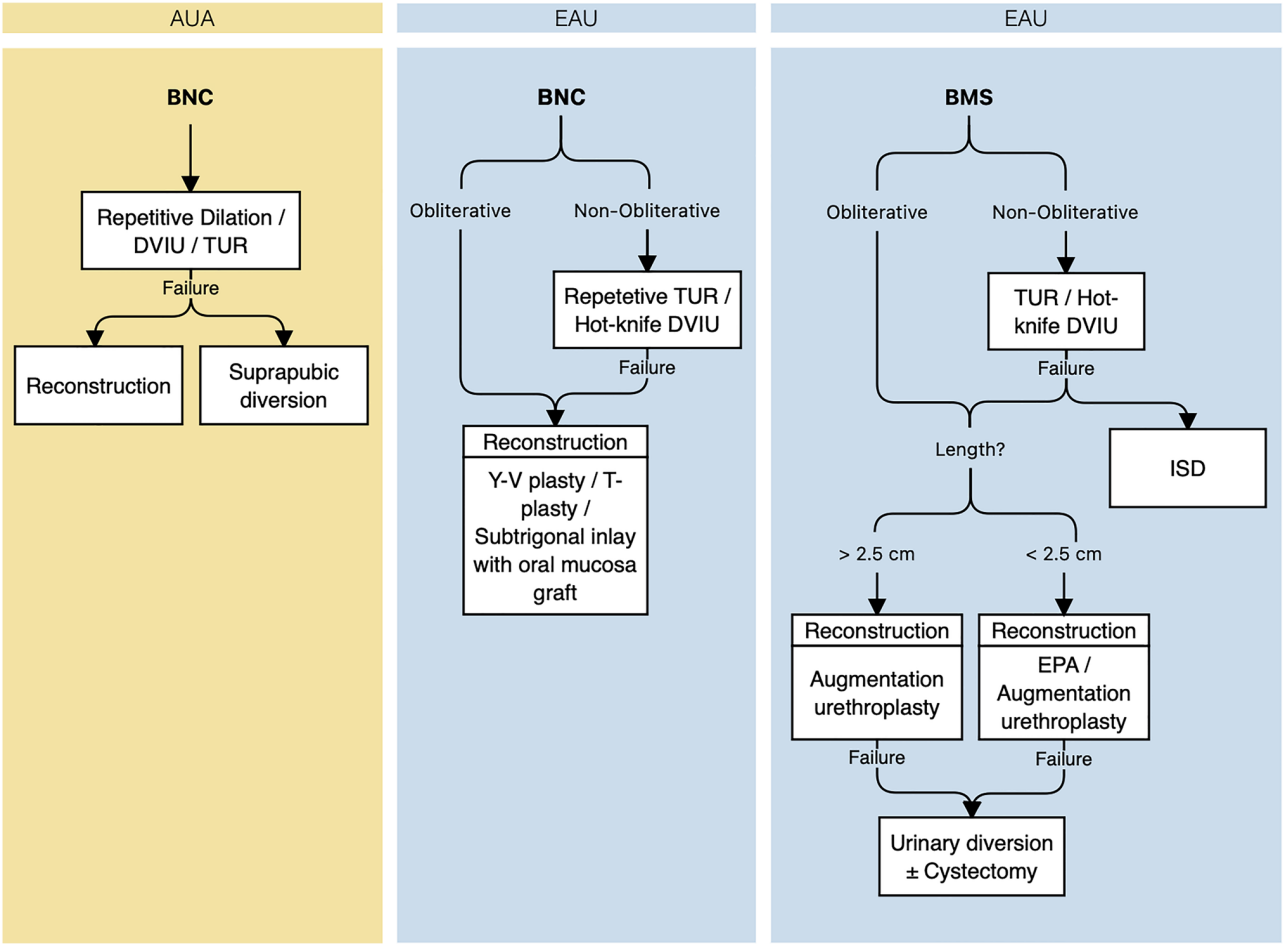
Guidelines on the management of posterior urethral obstruction due to surgical procedures for the management of benign prostate hyperplasia. AUA, American Urologic Association; EAU, European Urologic Association; BNC, Bladder neck contracture; BMS, Bulbomembranous stricture; DVIU, Direct-vision internal urethrotomy; TUR, trans-urethral resection; ISD, Intermittent self-dilation; EPA, Excision and primary anastomosis

In patients with non-obliterative stenosis in the bladder neck due to BPH surgeries, the surgeon should perform either trans-urethral resection (TUR) or hot-knife direct vision internal urethrotomy (DVIU) as the first-line modality [AUA: Expert opinion; EAU: Strong] (Fig. 1). Dilation could also be opted as the primary intervention according to AUA [AUA: Expert Opinion]; however, dilation is not included in EAU guidelines for BNC management (Table 1). In refractory cases, an open reconstruction of the bladder neck should be offered [AUA: Conditional; EAU: Weak] with Y-V or T-plasty [EAU: Weak]. Patients undergoing formal bladder neck reconstruction or repair of bulbo-membranous stricture (BMS) should be informed about the possibility of new-onset urinary incontinence [EAU: Strong].

**Table 1:** Summary of recommendations for the evaluation and management of urethral injuries due to pelvic fracture

	Shared	AUA	SIU	EAU
Diagnosis	Perform RUG. ^III, A, S^	–	Assess damage to the dual urinary sphincter mechanism. ^A^ Rule out urethral injuries in all pelvic fractures and highly suspect these injuries in pelvic ring disruption. ^B^ Avoid digital rectal examination. ^B^	Evaluate female urethral injuries with cystourethroscopy and vaginoscopy. ^S^
Initial management	Establish prompt urinary drainage in patients with PFUI. ^III, A, S^ Place SPC initially or after the failure of early urethral catheterization. ^A, S^	Initially, place SPC percutaneously or via open technique. ^III^ Place SPC in patients undergoing ORIF. ^V^	Immediately perform open primary repair and catheter realignment when concomitant bladder, BN, or rectal injuries are present. ^A^ SPC placement can be performed at emergency laparotomy if surgery is indicated for other injuries. ^A^	-
Early management	Perform realignment in stable patients (one attempt). ^III, B, W^ Avoid prolonged attempts / repeating at endoscopic realignment in PFUI cases. ^IV, S^ In general, avoid primary immediate urethroplasty. ^A, S^	-	Perform realignment by i) retrograde catheterization; ii) flexible cystoscope and retrograde passage of a guidewire; or iii) a combination of flexible and rigid cystoscopes passed antegrade/retrograde through a supra-pubic tract. ^B^ If successful, urethral catheterization should be maintained for 3 to 6 weeks. ^B^	Perform early urethroplasty (two days to six weeks) for men with complete disruption in selected patients (stable, short gap, soft perineum, lithotomy position possible). ^W^ Perform early repair (within seven days) for female patients (not delayed repair or early realignment). ^S^
Delayed management	-	-	Whenever possible, perform surgery. ^B^ For delayed reconstruction, the perineal midline progressive surgical approach is recommended. ^B^ Use standard or extended lithotomy position (extended lithotomy position should not take 5 h. <). ^B^ Indicators of the need for an elaborated perineal/trans-pubic repair include: length of defect/length of bulbar urethra > 0.35, a urethral gap length < 2.5 cm, and lateral prostatic displacement. ^B^ Perform inferior pubectomy prior to attempting BMA for any type of PFUI. ^B^ Perform supracrural rerouting in BMA when extra urethral length is required. ^B^ Consult about the post-operative incontinence in patients with a widely open BN with rectangular margins on cystography and with visible scarring on antegrade cystoscopy. ^B^ After successful urethroplasty via the perineum, postpone BN reconstruction (if necessary). In perineo-abdominal procedures, reconstruct BN in the same session. ^C^ For failed BMA, perform re-do BMA if the length of bulbar urethra is adequate. If not, penile skin flap (tubed) urethroplasty or staged urethroplasty using a perineal/abdominoperineal approach are the alternatives. ^B^	Manage complete posterior urethral disruption in male PFUIs with supra-pubic diversion and deferred (at least three months) urethroplasty. ^S^
Follow-up/complication management	-	Monitor patients for complications (e.g., stricture formation, erectile dysfunction, incontinence) for at least one year following urethral injury. ^III^	Use PDE5is for erectile function early after urethral injury. ^C^ Manage post-traumatic urethral fistula individually. ^B^ Manage post-traumatic incontinence individually by bladder neck repair/reconstruction, the artificial urinary sphincter, or a continent diversion. ^B^	–

*AUA* american urologic association, *EAU* european association of urology, *SIU* société internationale d’urologie, *RUG* retrograde urethrography, SPC supra-pubic catheter, *BN* bladder neck, *BMA* bulbo-membranous anastomotic urethroplasty

^−^no specific recommendation

^W^weak recommendation (EAU)

^S^strong recommendation (EAU)

^A^recommendation strength A (SIU)

^B^recommendation strength B (SIU)

^C^recommendation strength C (SIU)

^III^level of evidence C (AUA);

^IV^clinical practice (AUA)

^V^clinical principle (AUA)

### Posterior urethral stenosis following radiation/high energy therapies

#### BNC/Prostatic urethral stenoses

The most crucial consideration for bladder neck/prostatic urethral stenosis is whether there is a lumen. If there is no lumen, the stenosis is considered “obliterated”. The guidelines, particularly EAU, treat these as more severe cases with limited management options. For obliterative BNC, the EAU discourages endo-luminal procedures and early treatment with open reconstruction or diversion [EAU: Strong]. This recommendation and the strength of evidence are based on the near certainty of recurrence for endo-luminal treatment in this cohort.

For non-obliterative stenoses, the SIU recommends initial stenosis management with dilation, followed by cold/hot-knife DVIU or TUR combined with/without intermittent self-dilation (ISD) [SIU: C]. In case of primary DVIU/TUR failure, TUR should be performed/repeated once [SIU: C]. This differs from the EAU guidelines, which suggest a role for repetitive endo-luminal treatment for non-obliterative BNC to stabilize patency [EAU: Strong]. Growing evidence supports the efficacy of intralesional corticosteroids or mitomycin C injection during endo-luminal procedures to reduce the risk of recurrence in these patients [13–17]. However, no specific recommendation has been made by any of the guidelines in this regard.

**Fig. 2:**
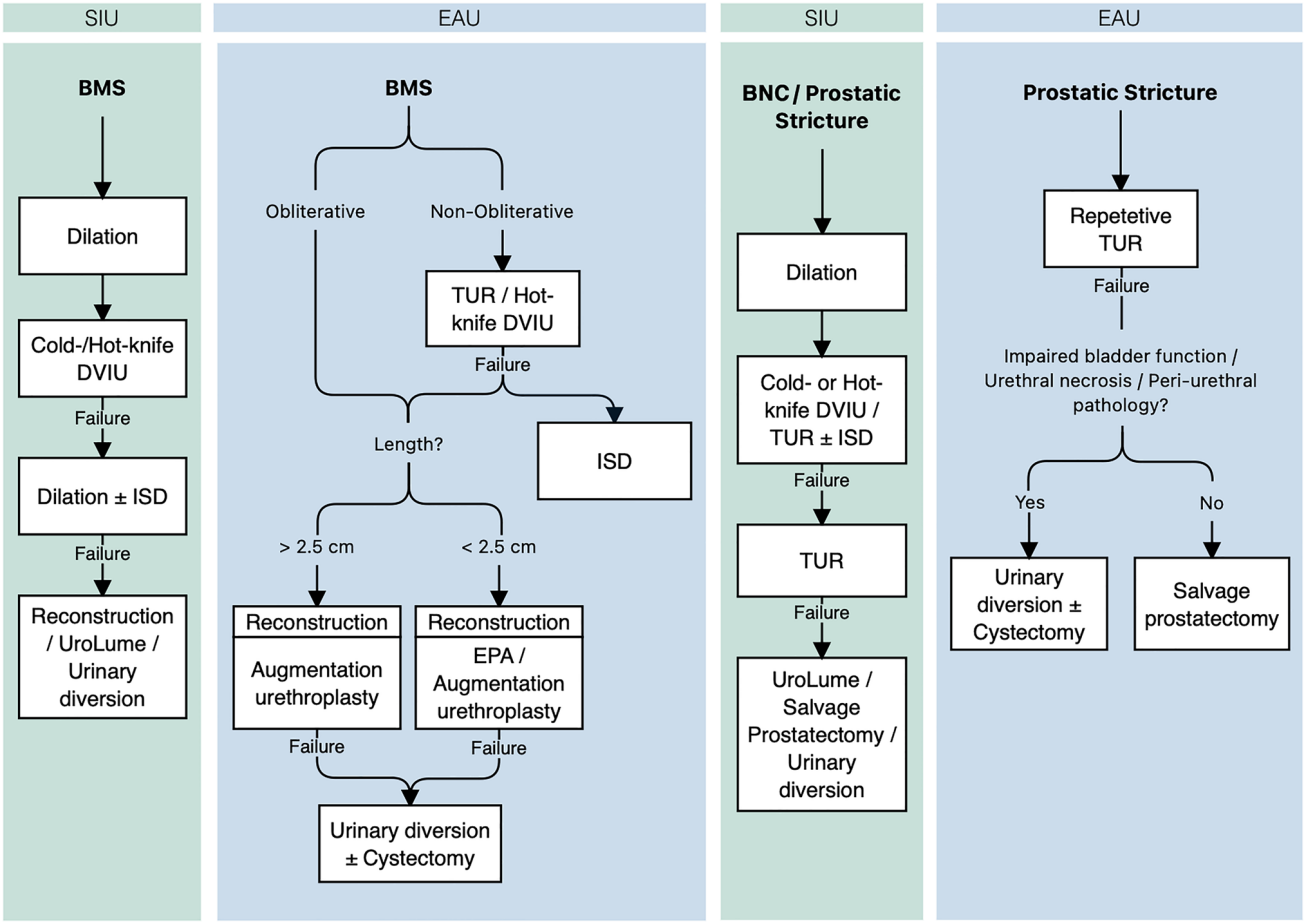
Guidelines on the management of posterior urethral obstruction due to pelvic irradiation. SIU, Société Internationale d’Urologie; EAU, European Urologic Association; BNC, Bladder neck contracture; BMS, Bulbomembranous stricture; DVIU, Direct-vision internal urethrotomy; TUR, trans-urethral resection; ISD, Intermittent self-dilation; EPA, Excision and primary anastomosis

The AUA and SIU agree on open reconstruction for recalcitrant radiation-induced BNC [AUA: Conditional; SIU: C], while the SIU uniquely offers posterior stenting (UroLume^®^) or supra-pubic/-vesical urinary diversion for non-urethroplasty candidates [SIU: C] (Fig. 2). Recently, robotic reconstruction for recalcitrant cases has been described, allowing for minimal incisions, reduced blood loss and post-operative pain, and shorter hospitalization and recovery [18–21]. Despite this, none of the guidelines have specifically addressed the use of robotic procedures for urethral stricture.

Lastly, salvage prostatectomy could be considered for medically fit patients with proper bladder function diagnosed with radiation-induced prostatic stricture [EAU: Weak].

#### Bulbomembranous/membranous strictures

For bulbo-membranous and membranous strictures, SIU suggests initial dilation, followed by cold-/hot-knife DVIU in case of failure [SIU: C], whereas EAU recommends either of the procedures for primary management of short non-obliterative radiation-induced BMS [EAU: Weak]. In completely obliterated strictures, EAU strongly opposes endoscopy and encourages augmentation urethroplasty [EAU: Strong]. When performing DVIU, the surgeon must avoid deep incisions at the six and twelve o’clock position [EAU: Strong] due to the risk of rectal injury and urosymphyseal fistula formation, respectively [6, 22]. According to SIU, DVIU could be repeated once combined with or without ISD [SIU: C]. Finally, in case of repeated DVIU failure, patients should be counseled on open reconstructive surgery [SIU: C; EAU: Weak], whether excision and primary anastomosis (EPA) or augmentation urethroplasty based on the skill and experience of the surgeon [EAU: Weak]. SIU recommends stenting (UroLume^®^) or supra-pubic/-vesical diversion in patients unfit for urethroplasty [SIU: C], while EAU discourages the use of stents in the posterior urethra due to low patency and proportionally high incontinence rates [23] [EAU: Weak]. The AUA offers no guidance as no endo-luminal stents are approved in the United States. Patients with radiation-induced BMS undergoing urethroplasty should be warned for de novo urinary incontinence and/or erectile dysfunction [EAU: Strong].

### Bladder neck contracture (BNC) due to radical prostatectomy

There is significant agreement between the guidelines on BNC management. Initial treatment should be with dilation or DVIU. Recurrent stenosis can be managed with repeat trans-urethral intervention. The SIU allows for the use of UroLume, which neither the AUA nor EAU recommend.

**Fig. 3:**
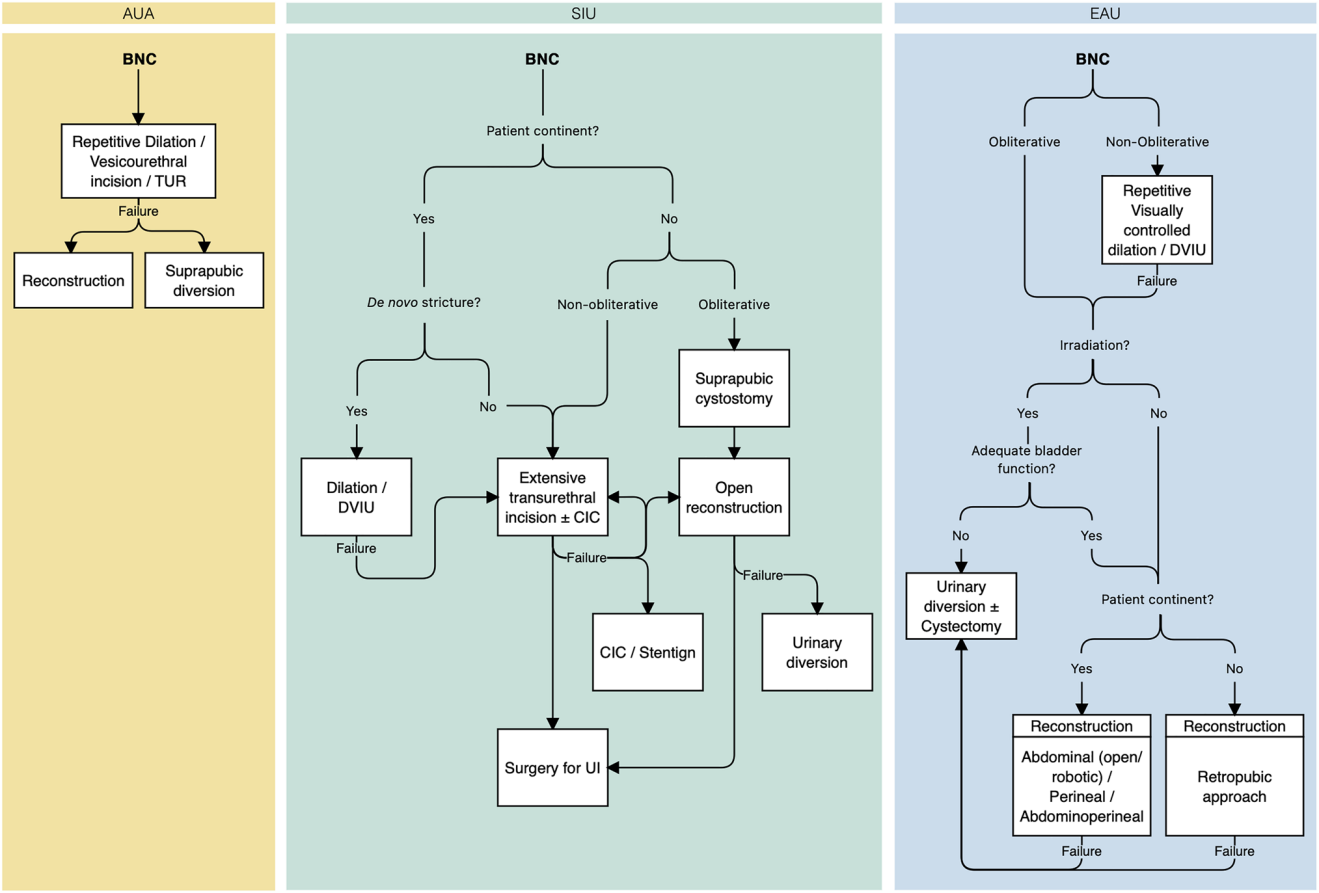
Guidelines on the management of bladder neck contracture due to radical prostatectomy. AUA, American Urologic Association; SIU, Société Internationale d’Urologie; EAU, European Urologic Association; BNC, Bladder neck contracture; DVIU, Direct-vision internal urethrotomy; TUR, trans-urethral resection; CIC, Clean intermittent catheterization; UI, Urinary incontinence

Similar to other etiologies, guidelines suggest avoiding endoscopic treatment of obliterative BNC and earlier open repair. For example, the SIU recommends the placement of a supra-pubic catheter (SPC) for drainage and open repair with no role for endo-luminal management. Similarly, the EAU and AUA recommend against endo-luminal treatment in the setting of obliterative stenosis. Revision of vesico-urethral anastomosis should be considered in non- and irradiated patients with functional bladder [AUA: Conditional; EAU: Weak] (Fig. 3). For incontinent patients, re-do vesico-urethral anastomosis could be approached retropubicly, and patients must be warned of the high risk of urinary incontinence if transperineal approach is being considered. According to SIU, urinary diversion should be considered for previously incontinent patients with obliterative stenosis.

Guidelines agree that urinary incontinence correction can be considered following resolution of obstruction, though the EAU discourages intervention prior to six months following re-do vesico-urethral anastomosis.

### Complex scenarios

For complex or recalcitrant stenosis, urinary diversion could be performed in patients with incapacitated bladder and/or disabling local symptoms [EAU: Weak]. Further, cystectomy could be considered in patients with incurable bladder pain/spasms and/or intractable hematuria [EAU: Weak].

## Trauma-related urethral stenosis

PFUI occurs in 1.6% to 25% of all pelvic fractures. However, the pelvic fractures leading to PFUI are significant, with 5% to 33% mortality [24]. Mechanistically, the term PFUI is applicable when the urethra is avulsed at the perineal membrane level, the point that membranous and bulbar urethras join [24, 25]. Avulsion may cause complete or partial urethral disruption and significant damage to the urethral sphincter [24, 26]. Given the extensive experience with traumatic posterior urethral injuries, we also include a short segment of the Urology Society of India Guidelines (USI) for this pathology.

According to AUA, the presentation of blood at the urethral meatus in patients with pelvic trauma indicates evaluation for PFUI (i.e., RUG). However, SIU suggests that even in the absence of classic signs of urethral injury (e.g., blood at the meatus and/or voiding difficulty) [24], fractures disrupting the pelvic ring are highly suggestive of concomitant urethral injuries [SIU: A]. Therefore, PFUI must be ruled out in all such pelvic fracture cases [SIU: A]. Palpable bladder and ‘high-riding prostate during digital rectal examination (DRE) are suggested as signs in favor of PFUI diagnosis [24]. However, SIU discourages the use of DRE as a diagnostic tool for PFUI, although it may be helpful in the evaluation of rectal injuries associated with pelvic fractures [SIU: B]. RUG is currently considered the gold standard for PFUI assessment [AUA; SIU: A]. An optimal RUG is performed by an experienced technician, envisioning the whole length of the urethra (including the bladder neck) to detect the site and stage of the injury [SIU: A]. EAU suggests flexible cystourethroscopy and/or RUG to evaluate male urethral injury and cystourethroscopy and vaginoscopy for the evaluation of female urethral injury. [EAU: Strong].

### Management

PFUIs could be managed either primarily or in a delayed fashion, implementing various techniques. While a full review of PFUI management is not in the scope of this review, those recommendations that have made it into guidelines are noteworthy as these procedures may prevent later stenoses.

#### Initial management

The guidelines unanimously recommend early bladder drainage as the patient could benefit through primary prevention of urinary extravasation and retention [24]. Drainage can be achieved by either supra-pubic or trans-urethral fashion, as it seems unlikely to induce additional damage to the urethra [SIU: A; EAU: Strong] [24]. However, while EAU does not consider either approach superior to the other, the SIU, AUA, and the USI guidelines name supra-pubic catheterization as the initial management of choice in most PFUI patients, which could be performed percutaneously under ultrasonographic guidance or during an emergent laparotomy [AUA: Expert opinion; SIU: A].

#### Early management

In patients with failed urethral catheterization or those treated with primary supra-pubic catheter placement, the guidelines generally favor early endoscopic urethral realignment after stabilizing hemodynamics. The early realignment minimizes extravasation of urine in partial injuries, while in complete defects, it is performed as an effort to reattach severely distracted ends rather than preventing the late-coming strictures [27]. The guidelines do not recommend open catheter realignment as it contains longer operations times and higher morbidities. Nevertheless, in case of the concomitant bladder neck or rectal injuries necessitating an open primary repair, SIU suggests performing simultaneous open realignment to avoid urinary incontinence or sepsis [SIU: A]. EAU and AUA, however, provide no specific recommendation.

A surgeon may opt for a variety of techniques for endoscopic realignment based on the surgeon’s expertise and the available instruments, ranging from simple retrograde catheterization to endoscopic procedures involving cystoscopy and retrograde guidewire passage or simultaneous use of flexible and rigid cystoscopes passed antegrade or retrograde. However, the clinicians must avoid prolonged endoscopic realignment attempts [AUA: Clinical Principle] as well as repeating endoscopic modalities following failure at realignment [EAU: Strong]. After successful urethral catheterization/realignment, the catheter should be maintained for 3 to 6 weeks [SIU: B]. There is some evidence to suggest that early endoscopic realignment may prevent stricture in future [28]. There are ongoing studies, including a randomized controlled trial, to evaluate the durability of this effect [29, 30].

Although immediate open urethroplasty should be avoided in patients with PFUI, EAU recommends early urethroplasty (from two days to six weeks) in selected patients who are stable and have a short gap between the urethral ends as well as soft perineum when the lithotomy approach is feasible [EAU: Strong]. Moreover, urethroplasty should be performed within seven days for female patients with PFUI, and realignment is not indicated [EAU: Strong].

#### Delayed management

Endoscopic dilation or DVIU can be attempted once for short non-obliterative stenoses [EAU: Weak]. Repetitive endoscopic treatments are associated with complications and likely not curative [31]. However, surgeons should choose urethroplasty over endoscopy if delayed management is being considered for completely obliterated stenoses due to pelvic fractures [AUA: Expert opinion; EAU: Strong] as endo-luminal treatments are primarily unsuccessful and may falsely pass through the bladder or rectum [6].

Reconstructive surgeries should be considered only after the stabilization of major accompanying injuries and performed by skilled surgeons in high volume centers, as it may not be feasible to anticipate the techniques required [AUA: Expert opinion; SIU: B; EAU: Weak]. However, to scheme the surgical procedure and approach, patients could undergo RUG with VCUG and/or antegrade/retrograde cystoscopy [AUA: Moderate]. VCUG studies revealing an open bladder neck with rectangular margins and observation of scarring during antegrade cystoscopy suggest internal sphincter damage for which the patient should be advised for post-operative urinary incontinence [SIU: B].

Technically, excision and primary anastomosis (EPA) should be considered for obliterative primarily, and non-obliterative stenoses after the failure of endo-luminal treatment [EAU: Strong]. The site of stenoses could be accessed via a midline perineal incision [SIU: B; EAU: Strong], for which the patient should be put in a standard or extended lithotomy position [SIU: B]. However, to avoid lower limb complications, surgeries with extended lithotomy must not take more than five hours [SIU: B]. Moreover, surgeons are expected to be proficient in inferior wedge pubectomy, which is considered an integral part of perineal bulbo-membranous EPA [SIU: B]. Further, in case of concomitant bladder neck injury, its repair should be postponed to a later session if a perineal approach is considered for bulbo-membranous reconstruction [SIU: C]. USI aligns with others in the recommendation of perineal approach with adequate scar excision and tension free bulbo-membranous anastomosis with rerouting reserved only when necessary. Combined abdominal-perineal approach may be required and should incorporate flap (ie omental) in such cases.

If bulbo-membranous EPA fails, the procedure could be repeated in fit patients not opting for urinary diversion or palliative care [EAU: Weak], provided that the length of the urethra is sufficient [SIU: B]; if not, single-staged or staged reconstruction using tubularized penile skin flap through a perineal or abdominoperineal approach could be performed [SIU: B]. USI also considers tubularized flaps in the setting of bulbar urethral necrosis. Further, adjunct supracrural rerouting is rarely suggested if the length of the urethra is not sufficient [SIU: B]. For recurrent short non-obliterative posterior stenosis after EPA, a maximum of two endo-luminal interventions (DVIU or dilation) could be performed if the urethral patency is considered long term [EAU: Weak].

In cases of ‘gapometry/urethrometry index’ —which is measured by dividing the length of the urethral gap by the length of the bulbar urethra— being below 0.35, a urethral gap over 2.5 cm, or prostatic lateral displacement, the progressive perineal approach may be insufficient, and the surgeon should consider elaborated perineal or trans-pubic repair [SIU: B]. However, one should reserve an abdominoperineal approach for more complicated scenarios, namely in cases of highly long distraction defect, para-urethral fistula at the base of the bladder, recto-urethral fistula related to trauma, and injury of the bladder [EAU: Weak]. Total pubectomy is discouraged during abdominoperineal repair [EAU: Strong], while bladder neck repair could be done simultaneously [SIU: C].

Post-traumatic urethral fistulas should be managed based on the location and etiology taking into account surgical expertise. The fistula should fully be exposed through a proper surgical approach, excised completely, and reconstructed with a well-vascularized flap [SIU: B]. For example, recto-urethral fistulas could be approached abdomino-perineally, and tissue flaps from the gracilis or rectus muscles could be used to fill the dead spaces [EAU: Weak].

### Complications

Post-traumatic urinary incontinence could be managed by urethral slings, artificial urinary sphincters, urinary diversion, or bladder neck reconstruction.

However, patient selection is of utmost importance [SIU: B]. Moreover, trauma-related ED may be alleviated with the early use of phosphodiesterase type 5 (PDE5) inhibitors [SIU: C]. More advanced treatment of ED may be required, particularly as many patients with PFUI are young. Finally, the potential damage to the internal and external sphincter mechanisms should always be evaluated in PFUIs [SIU: A].

## Conclusion

The recommendations provided by the three guidelines align considerably for the diagnosis, management, and follow-up of patients with posterior urethral stenosis. There remains a more prominent role for repeat endoscopic treatment in SIU and EAU guidelines compared to AUA. The preferred method to repair bulbo-membranous stricture/stenosis following radiation therapy remains an area of active interest, with a particular focus on continence preservation. Additionally, there may be a role for advanced endoscopic treatments with or without adjunct therapies to manage even obliterated stenoses.

## Supplementary Information

Below is the link to the electronic supplementary material.

Supplementary file1 (DOCX 30 KB)
