# Supplementary material for: Posterior urethral stenosis: a comparative review of the guidelines
Source: World J Urol. 2022 Aug 26;40(11):2591–600. doi: 10.1007/s00345-022-04131-y (PMC9617833; doi:10.1007/s00345-022-04131-y)
Supplement: Supplementary file 1 — Supplementary file1 (DOCX 30 KB) [file 345_2022_4131_MOESM1_ESM.docx]

**Appendix**

Comparison between the AUA, SIU, and EAU guidelines regarding (a) the level of evidence and (b) strength of recommendation.

| **a. Level of Evidence** | | |
| --- | --- | --- |
| **AUA** | **SIU** | **EAU** |
| **Grade A** (High quality evidence):  ﻿  well-conducted RCTs; exceptionally strong observational studies | **I** | **1a:**  SR of RCTs |
|  |  | **1b:**  Individual RCT with ‘good quality’ |
|  |  | **1c:**  ‘All-or-non’ studies |
| **Grade B** (Moderate quality evidence):  ﻿RCTs with some weaknesses; generally strong observational studies | **II** | **2a:**  SR of cohorts |
|  |  | **2b:**  Individual cohort study; low-quality RCT (<80% follow-up) |
|  |  | **2c:**  ‘Outcome’ research |
| **Grade C** (Low quality evidence):  **﻿**Observational studies providing conflicting information or with design  problems (e.g., very small sample size) | **III** | **3a:**  SR of case-control studies |
|  |  | **3b:**  Individual case-control study |
|  |  | **4:**  Case series; poor quality cohort; poor quality case-control |
| **-** | **IV** | **5:**  Expert opinions based on ‘first principles’ rather than evidence |
| **b. Recommendation Strength** | | |
| **AUA** | **SIU** | **EAU** |
| **Strong:**  **﻿**Benefits > Risks/Burdens  (Or vice versa)  **﻿**Net benefit (or net harm)  is/appears substantial  **Moderate:**  **﻿**Benefits > Risks/Burdens  (Or vice versa)  **﻿**Net benefit (or net harm)  Is/appears moderate  **Conditional:**  **﻿**Benefits=Risks/Burdens or unclear balance between Benefits &  Risks/Burdens  ﻿Best action (appears to)  depend on individual patient circumstances or alternative strategies may be equally reasonable  **Expert Opinion:**  **﻿**Statement about a component of clinical care widely agreed upon by urologists/other clinicians (no evidence)  **Clinical Principle:**  **﻿**Evidence-less statement, achieved by consensus of the Panel, that is based on members’ clinical  training, experience, knowledge (no evidence) | **A:**  **﻿**Usually consistent with level I evidence.  **B:**  **﻿**Consistent level II or III evidence or “majority evidence” from RCTs.  **C:**  **﻿**Level IV evidence or “majority evidence” from level II or III studies.  **D:**  **﻿**No recommendation (inadequate or conflicting evidence) | **Strong:**  Most patients would choose the recommended management; clinicians can structure their interactions with patients accordingly.  **Weak:**  Patients’ choices will vary according to their values and preferences; clinicians must ensure that patients’ care is in keeping with their values and preferences. |

*Abbreviations: SR, Systematic review; RCT, Randomized controlled trial.* Abbreviation: SR, Systematic review; RCT, Randomized controlled trial.
